# The difference of transcriptome of HPV-infected patients contributes more to the occurrence of cervical cancer than the mutations of E6 and E7 genes in HPV16

**DOI:** 10.1097/MD.0000000000036822

**Published:** 2024-01-19

**Authors:** Lihui Zhang, Mengyuan Li, Feiyan Yuan, Jingyuan Jiang, Xinmin Zhang

**Affiliations:** a Department of Gynaecology, The Second Norman Bethune Hospital of Jilin University, Changchun, China; b Department of Regenerative Medicine, School of Pharmaceutical Science, Jilin University, Changchun, China.

**Keywords:** cervical cancer, E6 gene, E7 gene, human papillomavirus, mutation

## Abstract

Human papillomavirus (HPV) E6 and E7 genes are biomarkers and drivers of the progression of cervical cancer (CxCa). The aim of this study was to investigate the relationship between HPV16 E6, E7 gene mutations and the occurrence and development of CxCa. Cervical exfoliated cells and clinical data of patients with cervical diseases were collected. Sample DNA was extracted, the E6 and E7 gene fragments were amplified by PCR, and the mutations were detected by Sanger sequencing and compared with standard sequences. Microarray was used to sequence the transcriptome of cells. Data of transcriptome analyzed and visualized using R software and its packages. Analysis of clinical characteristics demonstrated the association of HPV16 infection with CxCa (*P* < .05). Sanger sequencing results showed that the mutation sites of E6 gene included T178G/A, T350G, A131C, and T241G; among these, A131C and T241G were synonymous mutations. The mutation sites of E7 gene included A647G, T846C, G666A, T843C, and T760C, and all of them were synonymous mutations except A647G. There was no significant difference in the distribution of HPV16 E6, E7 mutations among CxCa, cervical intraepithelial neoplasia, and infection groups (*P* > .05). Compared with the non- CxCa group, gene ontology and Kyoto Encyclopedia of Genes and Genomes enrichment analysis of differentially expressed genes (DEGs) showed more significant enrichment of DEGs in the biological processes, pathways, and diseases closely related to cancer. Compared with the non-mutation group, the DEGs in the E6, E7 gene mutation group were significantly enriched in the events related to infection and immunity. To summarize, HPV16 may be associated with the occurrence and development of CxCa, but HPV16 E6 and E7 gene mutations have little effect on the occurrence and development of CxCa. Individual differences may have a greater effect on the progression of CxCa.

## 1. Introduction

According to the GLOBOCAN data compiled by the International Agency for Research on Cancer, cervical cancer (CxCa) is currently the fourth most common cancer in women. Approximately 604,000 new cases of CxCa were diagnosed worldwide in 2020 and there were approximately 342,000 deaths attributed to CxCa in the same year.^[1]^ Human papillomavirus (HPV) infection is the main risk for CxCa and plays a key role in the development of cervical lesions and CxCa. Persistent HPV infection may eventually develop into CxCa.^[2,3]^ HPV infection begins in the basal layer of the stratified squamous epithelium, where the virus replicates initially at a low copy number; when the basal cells differentiate to form the basal layer of the epithelium, the viral genome switches to a high-copy mode, and the virus particles are subsequently released upon epithelial exfoliation, leading to infection of the neighboring cells.^[4]^ After the integration of the viral genome with the host genome, HPV E6 and E7 early proteins are responsible for the replication of the viral genome, and these can induce cells to transform into cancer cells during the process of viral replication.^[5,6]^ According to its pathogenic ability after infecting skin epithelial cells or inner tissues, HPV can be divided into low-risk and high-risk types.^[7]^ Some low-risk HPV viruses can cause benign cervical lesions and genital warts, while high-risk HPV plays a key role in the occurrence and development of CxCa.^[1]^ Infection with high-risk HPV is a commonly observed feature in many patients with CxCa.^[8]^ Among the high-risk types, HPV16 of mucosal type α is the most frequently detected genotype in 60.5% of CxCa and is classified as a class I carcinogen.^[9]^

The gene sequence of HPV16 consists of 6 early open reading frames (E1, E2, E4, E5, E6, E7), 2 late reading frames (L1, L2) and a long noncoding control region.^[10]^ Among them, the early genes (E1, E2, E4, E5, E6, E7) of HPV16 are the key genes that regulate its replication and pathogenicity.^[11]^ E1 is an ATP-dependent DNA helicase which is essential for the replication and amplification of viral fragments in infected cells.^[12]^ E2 plays an important role in DNA replication and post-transcriptional processing and packaging.^[13]^ The open reading frame of E4 is contained in E2, while E5 may play a role in the life cycle of HPV16 virus.^[14]^ E6 and E7 are responsible for several cellular checkpoints that may induce the initiation of genital tumors, including CxCa.^[10]^ HPV E6 and E7 viral oncoproteins induce malignant proliferation, angiogenesis, invasion, migration, and unrestricted telomerase activity, as well as apoptosis of unaffected cells and growth inhibitor activity during viral genome replication.^[15,16]^ E6 binds the p53 tumor suppressor through ubiquitin ligase E6-AP and pro-apoptotic Bcl2 protein and inhibits pro-caspase-8 activation to prevent apoptosis, thereby forcing cells to evade preventive checkpoints through uncontrolled cell division.^[17,18]^ E7 targets pRb for ubiquitination, leading to the release of E2F transcription factors and forcing cells to enter S phase in advance, thus enabling cells to pass the G1-S forced checkpoint and achieve unlimited cell proliferation.^[19]^

Because of the close relationship of E6 and E7 gene expression with CxCa, the relationship between the mutations of these genes and CxCa has attracted more attention. Although the expression of E6 and E7 genes is believed to be most closely related to the occurrence of CxCa,^[10]^ studies have identified many lineages of mutations in E6 and E7 gene sequences, including European (EUR), Asian (As), African 1 (AFR1), African 2 (AFR2), and North American/AsiAn-American (NA/AA).^[20]^ Different genetic mutations have different effects on the development of CxCa. Some studies on E6 gene C287G mutation, non-European lineage mutation, and EP/EA lineage mutation found that HPV16 gene mutation altered the biological function of the protein encoded by the mutant gene; this phenomenon may affect the persistence of infection and the incidence of malignant transformation of cervical lesions.^[21–23]^ However, in a study conducted in Indonesian women, HPV16 E6 and E7 gene mutations showed no correlation with the expression level of tumor suppressor p53.^[24]^ In addition, EP[a], the most common variant found in a large clinical study of 10,000 women in Costa Rica, also showed no association with CxCa.^[25]^

To summarize, HPV16 infection and its own gene mutations are closely related to the occurrence and development of CxCa, but there is no clear consensus on the correlation of E6 and E7 gene mutations with the occurrence and development of CxCa. Therefore, in this study, we analyzed the relationship of HPV16 infection and its E6 and E7 gene mutations with the clinical features and whole genome transcriptome. Our findings suggest that after HPV infection, the inter-individual differences in gene expression of patients themselves may play a more important role than the mutations in E6 and E7 genes of HPV16.

## 2. Materials and methods

### 2.1. Samples collection

After obtaining written informed consent of patients and review by Ethics Committee of the Second Bethune Hospital of Jilin University, 218 Ziqiang Street, Nanguan District, Changchun City, Jilin Province (Approval No. 20190101000033), samples from 120 patients presented to the Second Hospital of Jilin University with cervical diseases were collected from August 2019 to December 2021, The age range of the patients was 19 to 65 years, the mean and average age of the sample was 43. Among these, 95 patients with HPV positivity were divided into CxCa group and cervical intraepithelial neoplasia (CIN group), and 25 patients with HPV positivity but normal cytology or negative cervical biopsy were classified as the infection group.

The inclusion criteria for samples were: cervical exfoliated cells from HPV-positive patients; no recent local or systemic antiviral treatment; no history of treatment for cervical disease. The exclusion criteria were: cervical exfoliated cells from HPV-negative patients; history of other malignant tumors, autoimmune diseases, or history of receiving oral immunosuppressants.

No vaginal irrigation, vaginal medication, or sexual intercourse were performed during the 3-day period immediately preceding sample collection, and the menstrual period was avoided. Cervical exfoliated cells were collected with a sterile disposable cervical sampler at the time of biopsy collection. The diagnoses of CxCa and CIN were based on histopathological examination of cervical biopsy tissue (obtained via colposcopy) or surgically resected cervical tissue. In patients classified as the infection group, cervical liquid-based cytology showed no intraepithelial lesion or malignancy or colposcopic cervical biopsy showed no cervical lesions. The staging of CxCa was based on the 2018 International Federation of Gynecology and Obstetrics (FIGO) surgical staging system. The patient name, age, gravidity, parity, clinical stage, pathological grade, HPV infection type, and other clinical information were collected. HPV infection typing, HPV16 positivity, and other types of infection were determined by alignment of PCR-amplified L1 sequences.

### 2.2. Sample DNA extraction and identification

The collected cells were processed according to the instructions of ONE-4-ALL genomic DNA extraction kit provided by Shanghai Bioengineering Co. Ltd., and the DNA in the samples was extracted.

### 2.3. Amplification and sequencing of HPV16 E6 and E7 sequence

E6 and E7 amplification primer upstream: 5′- GTTGAACCGAAACCGGTTAG-3′, downstream: 5′-TCTCTGTTTCTGCCTGTGTT-3′, respectively. The primers were synthesized by Shanghai Bioengineering Co. Ltd.

The PCR system was 0.5 μL of upstream and downstream primers, 1 μL of DNA, 10.5 μL of distilled water, and 12.5 μL of Premix Taq1. The PCR reaction program was as follows: 1 cycle at 95ºC for 3 minutes, 34 cycles at 95ºC for 30 seconds, 45ºC (each cycle was increased by 0.1ºC) for 1 minute, and 72ºC for 2 minutes, 1 cycle at 72ºC for 5 minutes. After detection by agarose gel electrophoresis, the clear single-band PCR product samples were labeled and sent to Shanghai Bioengineering Co. Ltd. for sequencing. And the primers used for E6 and E7 sequencing were 5′-GTTGAACCGAAACCGGTTAG-3′ and 5′-CATTTTCGTTCTCGTCATCTG-3′.

### 2.4. Transcriptome microarray

Thirty-two samples were randomly selected from 120 samples and sent to Puchuan Pharmaceutical Co. Ltd. of Jilin Province for detecting differentially expressed genes (DEGs) in the whole genome using microarray.

### 2.5. Data processing

The relationship between HPV infection and clinical features was analyzed using EXCEL and R software (v4.2.2). The results of first generation sequencing were compared with HPV16 standard sequence by SeqMan (v7.1) and SnapGene (v6.0.2) software in DNAstar software to determine whether the samples were HPV16 infected and to identify the E6, E7 mutation sites. The results of chip sequencing were screened for DEGs using the EdgeR package of R language (v4.2.2). Clusterprofile package was used for gene ontology (GO) and Kyoto Encyclopedia of Genes and Genomes (KEGG) enrichment analysis. The ggplot2 package was used for visualization. Each experiment was repeated at least 3 times. Analysis of chi-square test and Fisher exact test were used to analyze the data according to different types. *P* < .05 was considered statistically significant.

## 3. Results

### 3.1. HPV16 positive infection is associated with CxCa

The results of HPV16 infection test in 120 patients are shown in Table 1. As assessed by chi-square test, the distribution of the number of HPV16 positive patients in CxCa group, CIN group, and infection group was significantly different (χ2 = 14.348, *P* = .001). The proportion of HPV16 positive cases increased with the degree of CxCa lesions.

**Table 1:** HPV16 infection test results.

	HPV16+	HPV16−	Total	
Cervical cancer group	29 (73.68)	9 (26.32)	38	*χ*^2^ = 14.348 *P* = .001*
CIN group	32 (56.14)	25 (43.86)	57	
Infection group	7 (28.00)	18 (72.00)	25	
Totally	68	52	120	

Data presented as frequency (%).

CIN = cervical intraepithelial neoplasia, HPV = human papillomavirus.

* *P* < .001, HPV16 + vs HPV16−.

### 3.2. HPV16 E6 gene mutation is not associated with CxCa

In the CxCa group, there were 19 cases with E6 gene mutation; 10 base mutation sites and a total of 8 kinds of amino acid changes were found. Among them, T178G/A, T350G, A131C, and T241G were found to be mutated more than twice. The corresponding amino acid changes of these 4 base mutation sites were D25E, L83V, R10R, and A46A, among which the 2 base mutation sites A131C and T241G were synonymous mutations, which did not cause amino acid change. In the CIN group, there were 19 cases with E6 gene mutation, and 8 base mutation sites were found. The sites with mutation frequency ≥ 2 times were T178G/A and A131C, and A131C was nonsense mutation. In the simple infection group, there were 4 cases of E6 gene mutation, including 3 cases of T178G/A and 1 case of T350G. As assessed by Fisher exact test, there was no association in the distribution of these 4 mutation sites among the 3 groups (*P* > .05) (Table 2).

**Table 2:** Statistics of HPV16 E6 gene mutation.

Mutation site	Cervical cancer group (n = 29)	CIN group (n = 32)	Infection group (n = 7)	*P* value
T178G/A	14 (48.28)	16 (50.00)	3 (42.86)	>.99
T350G	2 (6.90)	1 (3.13)	1 (14.29)	.31
A131C	2 (6.90)	3 (9.38)	0	>.99
T241G	2 (6.90)	1 (3.13)	0	.71

Data presented as frequency (%).

CIN = cervical intraepithelial neoplasia.

### 3.3. HPV16 E7 gene mutation is not associated with CxCa

In the CxCa group, there were 24 cases of E7 gene mutations, and a total of 8 base mutation sites were found, resulting in 2 kinds of amino acid substitutions. Among these, 5 base mutation sites were mutated more than twice each: A647G, T846C, G666A, T843C, T760C; the corresponding amino acid changes of these 5 base mutation sites were N29S, S95S, E35E, C94C, and L67L. Except for A647G, the other mutation sites were synonymous mutations, which did not cause amino acid change. A total of 25 samples in the CIN group had E7 gene mutations, and a total of 8 base mutation sites were found, resulting in 2 kinds of amino acid substitutions. Among these, 5 base sites with mutation frequency ≥ 2 times were: A647G, T846C, G666A, T843C, and T760C. These were synonymous mutations, except A647G, which did not cause amino acid change. In the simple infection group, E7 gene mutations were found in 4 cases, including A647G and T846C mutations in 2 cases, G666A mutation in 1 case, and T843C mutation in 1 case. Statistical analysis showed co-existence of A647G and T846C mutations, i.e., there may be a co-mutation phenomenon. However, as assessed by Fisher exact test, there was no association in the distribution of these 5 mutation sites among the 3 groups (*P* > .05) (Table 3).

**Table 3:** Statistics of HPV16 E7 gene mutation.

Mutation site	Cervical cancer group (n = 29)	CIN group (n = 32)	Infection group (n = 7)	*P* value
A647G	12 (41.38)	15 (46.88)	2 (28.57)	.73
T846C	12 (41.38)	15 (46.88)	2 (28.57)	.73
G666A	9 (31.03)	8 (25.00)	1 (14.29)	.79
T843C	4 (13.79)	5 (15.63)	1 (14.29)	>.99
T760C	2 (10.53)	2 (6.25)	0	>.99

Data presented as frequency (%).

CIN = cervical intraepithelial neoplasia.

### 3.4. HPV16 E6, E7 gene mutations showed no association with clinical features of CxCa

As assessed by Fisher exact test, in CxCa patients, HPV16 E6 and HPV16 E7 gene mutations showed no significant association with FIGO stage, pathological type, vascular metastasis, or lymph node metastasis (*P* > .05) (Tables 4 and 5). Detailed clinical features is provided in Table S1, Supplemental Digital Content, http://links.lww.com/MD/L266.

**Table 4:** Association of HPV16 E6 gene mutations with clinicopathological features of cervical cancer.

	E6 mutations	E6 unmutated	*P* value
Total n	19	10	
FIGO stage n (%)			
I	14 (73.7)	9 (90.0)	.35
II	1 (5.3)	1 (10.0)	
III	4 (21.1)	0 (0.0)	
Pathological type n (%)			
Squamous carcinoma	15 (78.9)	9 (90.0)	>.99
Adenocarcinoma	3 (15.8)	1 (10.0)	
Adenosquamous carcinoma	1 (5.3)	0 (0.0)	
Vascular metastasis n (%)			
+	9 (47.4)	5 (50.0)	>.99
−	9 (47.4)	5 (50.0)	
Unknown	1 (5.3)	0 (0.0)	
Lymph node metastasis n (%)			
+	5 (26.3)	2 (20.0)	>.99
−	13 (68.4)	8 (80.0)	
Unknown	1 (5.3)	0 (0.0)	

FIGO = International Federation of Gynecology and Obstetrics.

**Table 5:** Association of HPV16 E7 gene mutations with clinicopathological features of cervical cancer.

	E7 mutations	E7 unmutated	*P* value
Total n	24	5	
FIGO stage n (%)			
I	19 (79.2)	4 (80.0)	.42
II	1 (4.2)	1 (20.0)	
III	4 (16.7)	0 (0.0)	
Pathological type n (%)			
Squamous carcinoma	20 (83.3)	4 (80.0)	.37
Adenocarcinoma	3 (12.5)	0 (0.0)	
Adenosquamous carcinoma	1 (4.2)	1 (20.0)	
Vascular metastasis n (%)			
+	13 (54.2)	1 (20.0)	.44
−	10 (41.7)	4 (80.0)	
Unknown	1 (4.2)	0 (0.0)	
Lymph node metastasis n (%)			
+	7 (29.2)	0 (0.0)	.41
−	16 (66.7)	5 (100.0)	
Unknown	1 (4.2)	0 (0.0)	

FIGO = International Federation of Gynecology and Obstetrics.

### 3.5. Transcriptome of patients affects cervical carcinogenesis

**Figure 1.**
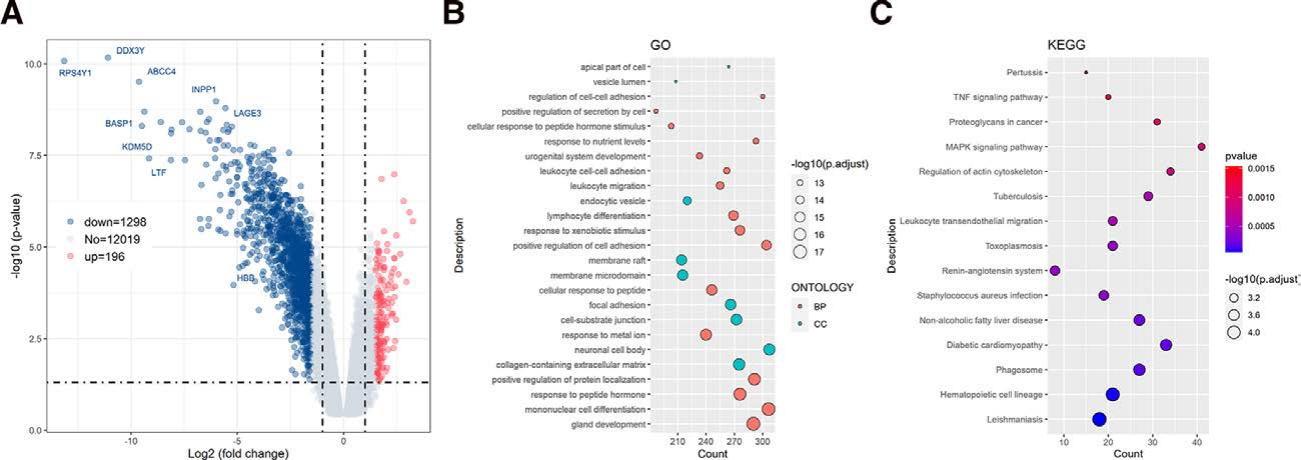
Effect of patient gene expression on cervical carcinogenesis. (A) Volcano plot of differential gene expression. Red indicates up-regulated genes, and blue indicates down-regulated genes. The horizontal dashed line indicates *P* = .05, and the vertical dashed lines indicate log2FC = +1.5 and log2FC = −1.5; (B) GO enrichment analysis of DEGs. Blue represents BP, orange represents CC; (C) KEGG pathway enrichment analysis of DEGs. The abscissa represents the count of enriched genes, and the ordinate represents pathways. The color gradient from blue to red indicates the change in *P* value from small to large after correction. BP = Biological process, CC = cellular component, DEGs = differentially expressed genes, GO = gene ontology, KEGG = Kyoto Encyclopedia of Genes and Genomes.

The 32 sequencing samples were divided into CxCa group and non- CxCa group for sequencing data analysis and comparison. The volcano map of DEGs is shown in Figure 1A. Using the *P* < .05 and | log2FC |≥1.5 standard, 196 up-regulated genes and 1298 down-regulated genes were identified. GO enrichment analysis of DEGs (Fig. 1B) showed that biological process (BP) was mainly enriched in gland development, mononuclear cell differentiation, response to peptide hormones, and positive regulation of protein localization, and cellular component (CC) was mainly enriched in collagen-containing extracellular matrix, neuronal cell body, and cell-substrate junction. The results of KEGG enrichment analysis (Fig. 1C) showed that the DEGs were mainly enriched in leishmaniasis, hematopoietic cell lineage, phagosome and diabetic cardiomyopathy.

**Figure 2.**
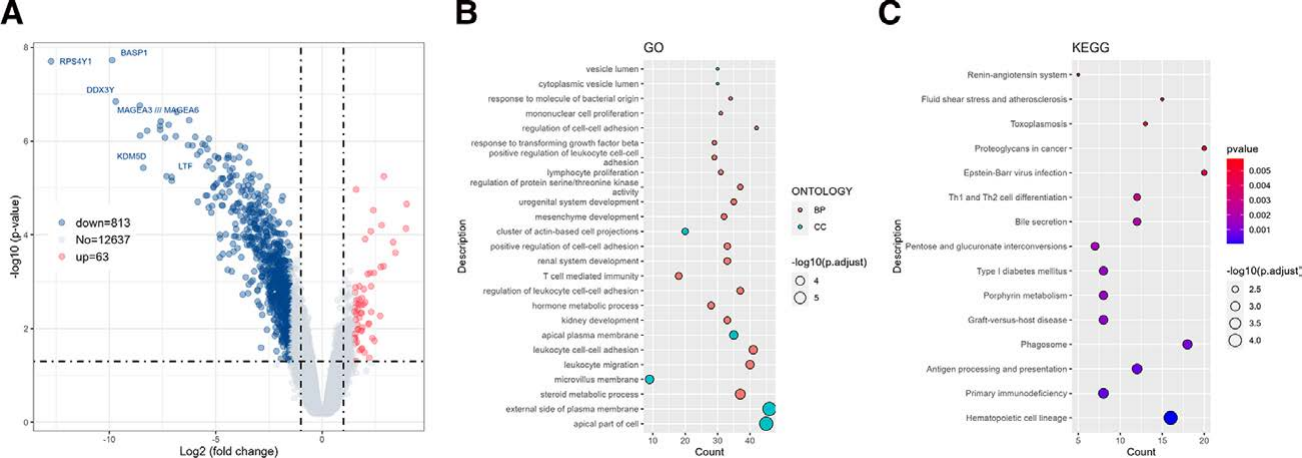
Effect of HPV16 E6 gene mutation on cervical carcinogenesis. (A) Volcano plot of differential gene expression. Red indicates up-regulated genes, and blue indicates down-regulated genes. The horizontal dashed line indicates *P* = .05, and the vertical dashed lines indicate log2FC = +1.5 and log2FC = −1.5; (B) GO enrichment analysis of DEGs. Orange represents BP, green rep-resents CC; (C) KEGG pathway enrichment analysis of DEGs. The abscissa represents the count of enriched genes, and the ordinate represents pathways. The color gradient from blue to red indicates change in *P* value from small to large after correction. BP = Biological process, CC = cellular component, DEGs = differentially expressed genes, GO = gene ontology, KEGG = Kyoto Encyclopedia of Genes and Genomes.

Then the sequencing samples were divided into HPV16 E6 gene mutation group and non-mutation group for sequencing data analysis and comparison. The volcano map of DEGs is shown in Figure 2A. Using the *P* < .05 and | log2FC |≥1.5 standard, 63 up-regulated genes and 813 down-regulated genes were identified. GO enrichment of DEGs (Fig. 2B) showed that BP was mainly enriched in steroid metabolic process, leukocyte migration, leukocyte cell-cell adhesion and kidney development; CC was mainly enriched in apical part of cell, external side of plasma membrane and micro-villus membrane. KEGG enrichment analysis (Fig. 2C) showed that the DEGs were mainly enriched in hematopoietic cell lineage, primary immunodeficiency, antigen processing and presentation and phagosome.

**Figure 3.**
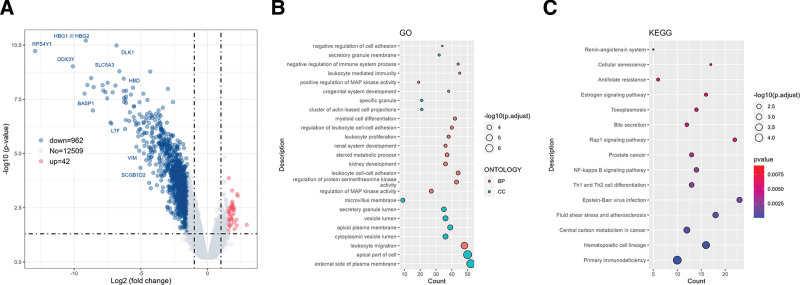
Effect of HPV16 E7 gene mutation on cervical carcinogenesis. (A) Volcano plot of differential gene expression. Red indicates up-regulated genes, and blue indicates down-regulated genes. The horizontal dashed line indicates *P* = .05, and the vertical dashed lines indicate log2FC = +1.5 and log2FC = −1.5; (B) GO enrichment analysis of DEGs. Orange represents BP, green represents CC; (C) KEGG pathway enrichment analysis of DEGs. The abscissa represents the count of enriched genes, and the ordinate represents pathways. The color gradient from blue to red indicates change in *P* value from small to large after correction. BP = Biological process, CC = cellular component, DEGs = differentially expressed genes, GO = gene ontology, KEGG = Kyoto Encyclopedia of Genes and Genomes.

Last the sequencing samples were divided into HPV16 E7 gene mutation group and non-mutation group for sequencing data analysis and comparison. The volcano map of DEGs is shown in Figure 3A. Using *P* < .05 and | log2FC |≥1.5 standard, 42 up-regulated genes and 962 down-regulated genes were identified. GO enrichment of DEGs (Fig. 3B) showed that BP was mainly enriched in leukocyte migration, regulation of MAP kinase activity, regulation of protein serine/threonine kinase activity and leukocyte cell-cell adhesion; CC was mainly enriched in external side of plasma membrane, apical part of cell and cytoplasmic vesicle lumen. KEGG enrichment analysis (Fig. 3C) showed that the DEGs were mainly enriched in primary immunodeficiency, hematopoietic cell lineage, central carbon metabolism in cancer and fluid shear stress and atherosclerosis.

## 4. Discussion

Both genetic and environmental factors are implicated in the causation of CxCa.^[26]^ Persistent infection with high-risk HPV is one of the main causes of CxCa.^[27]^ Integration of high-risk HPV genome into cellular chromatin can promote the occurrence of CxCa.^[28]^ HPV16 is the most pathogenic type of high-risk HPV causing CxCa.^[29]^ In the present study, the proportion of HPV16 positive cases increased with the severity of CxCa lesions, indicating that HPV16 infection is indeed related to the progression of CxCa lesions and may promote the development of CxCa.

The evolution of human beings and the emergence of regional differences have led to increased frequency of naturally occurring genetic mutations of HPV16. Based on the differences between the L1 coding region of the DNA sequence of a new HPV type and the existing prototype, new HPV types or the same genotype variant lineages and sublineages can be defined.^[30–32]^ Studies conducted in Sweden and Denmark have demonstrated the association of HPV16 gene variants with persistent HPV infection and progression of CxCa lesions.^[33,34]^ However, some studies have yielded inconsistent results. In the French and British populations dominated by HPV16 European variants, there was no significant association of HPV16 variants with persistent HPV infection and cervical disease progression.^[35,36]^ Another study found no correlation of HPV16 E6 and E7 gene mutations with the expression level of tumor suppressor p53 in Indonesian women.^[24]^ Moreover, EP[a], the most common variant identified in a clinical study of 10,000 women in Costa Rica, also showed no association with CxCa.^[25]^ In this study, T178G/A, T350G, A131C, and T241G were the most frequent mutations of HPV16 E6 gene. A131C and T241G were synonymous mutations, which did not cause amino acid change. There were 5 base sites of HPV16 E7 gene mutation more than twice, including A647G, T846C, G666A, T843C, and T760C. Except for A647G, the base mutation sites were all synonymous mutations, which did not cause amino acid change. There was no significant difference in the distribution of these mutation sites among the 3 groups with different degrees of cervical lesions (*P* > .05), indicating a lack of association of HPV16 E6 and E7 gene mutations with CxCa. A study on the variants of HPV16 in northeast China found T178G as the most common mutation site in E6, and A647G as the most common mutation site in E7,^[37]^ which was consistent with our results. However, in this study, there was no significant association of HPV16 E6, E7 mutation with FIGO stage, pathological type of CxCa, vascular metastasis, or lymph node metastasis, which also indicated that HPV16 E6, E7 gene mutations were not significantly associated with the occurrence and development of CxCa. This may be due to the fact that a large proportion of these mutations are synonymous mutations, which do not cause amino acid changes; therefore, despite the occurrence of gene mutations, it showed a weak relation with the occurrence and progression of CxCa. Recent studies have also shown a low proportion of non-synonymous mutations in E7 in CxCa, while precancerous lesions showed a high mutation rate of E7 gene; this suggests that E7 gene mutation may reduce the oncogenicity of HPV16. This implies that the oncogenicity of HPV16 depends on the highly conserved E7 protein.^[38]^ In this study too, E7 showed a higher mutation rate than E6, but most of the mutations were synonymous mutations. The frequency of A647G mutation in the CIN group was slightly higher than that in the CxCa group, but the difference was not statistically significant due to the small sample size.

In high-risk HPV infection such as HPV16, E6 and E7 gene mutations may affect the malignant transformation process, including cell cycle arrest and loss of apoptosis caused by E6 and transitional proliferation caused by E7.^[39–41]^ E6 and E7 oncoproteins are tumor specific and tumor rejection antigens, which are expressed in tumors and precursor lesions and are ideal targets for immunotherapy.^[42]^ Moreover, E6 and E7 proteins contribute to viral immune escape and promote tumor development by interacting with a variety of proteins.^[43]^ In this study, the DEGs between HPV16 E6 gene mutation group and non-mutation group, and the DEGs between HPV16 E7 gene mutation group and non-mutation group were more enriched in hematopoietic cell lineage and primary immunodeficiency in KEGG enrichment analysis. This indicates that the mutations of E6 and E7 gene may be less related to the occurrence of CxCa. However, these are related to the occurrence of immune-related events, which is consistent with the previous studies mentioned above.^[24,25,35,36]^ The KEGG enrichment analysis of DEGs between CxCa group and non- CxCa group showed that these DEGs were enriched in MAPK signaling pathway. This indicates that the occurrence of CxCa may be related to the activation of MAPK signaling pathway, which is also consistent with a previous study that implicated the MAPK signaling pathway in the development of CxCa.^[44]^ GO enrichment analysis of these DEGs showed that the occurrence of CxCa was mainly related to gland development, mononuclear cell differentiation, response to peptide hormones, positive regulation of protein localization, collagen-containing extracellular matrix, neuronal cell body, cell-substrate junction, and other BPs. This suggests that the occurrence of CxCa may be closely related to the gene expression of patients themselves and individual differences.

To conclude, this study found no evidence that HPV16 E6 and E7 gene mutations are related to the occurrence and development of CxCa. Although high-risk HPV16 infection can promote the occurrence and development of CxCa, the effect of HPV16 gene mutation on the progression of cervical lesions appears to depend on individual biological differences and gene mutation types. However, due to the small number of samples collected in this study, some of the between-group differences were not statistically significant, and some HPV16 gene mutations only appeared once. Thus, the effect of these mutations on the occurrence and development of CxCa could not be analyzed. Therefore, a larger study is required to further investigate the effect of different HPV16 gene mutations on the occurrence and development of CxCa and its related mechanism.

## Author contributions

**Conceptualization:** Lihui Zhang, Mengyuan Li, Feiyan Yuan, Jingyuan Jiang, Xinmin Zhang.

**Data curation:** Feiyan Yuan.

**Formal analysis:** Lihui Zhang, Mengyuan Li, Feiyan Yuan, Jingyuan Jiang, Xinmin Zhang.

**Funding acquisition:** Lihui Zhang.

**Methodology:** Lihui Zhang, Mengyuan Li, Feiyan Yuan, Jingyuan Jiang, Xinmin Zhang.

**Supervision:** Xinmin Zhang.

**Writing – original draft:** Lihui Zhang, Mengyuan Li, Xinmin Zhang.

**Writing – review & editing:** Lihui Zhang, Mengyuan Li, Xinmin Zhang.

## Supplementary Material


